# Acupuncture Improving Early Sexual Development of Girls with Peripheral Precocious Puberty: A Prospective Cohort Study

**DOI:** 10.1155/2020/8091846

**Published:** 2020-04-10

**Authors:** Lili Liu, Naijun Wan, Huihui Sun

**Affiliations:** ^1^Acupuncture Department, Beijing Jishuitan Hospital, Beijing 100035, China; ^2^Pediatrics Department, Beijing Jishuitan Hospital, Beijing 100035, China

## Abstract

**Objective:**

To study primarily on the effect of acupuncture on breast Tanner stage, serum sex hormone level, and TCM symptom scores in girls with peripheral precocious puberty (PPP).

**Methods:**

19 eligible patients diagnosed with PPP received acupuncture intervention for 12 weeks, twice a week for 12 weeks, 24 sessions of acupuncture treatment in all, and then follow-up for 12 weeks. The primary outcome was the change in serum luteinizing hormone (LH) level, follicle-stimulating hormone (FSH) level, and breast Tanner stage at 12-week treatment and 12-week follow-up. Serum estradiol (E2) level and TCM symptom scores were also assessed.

**Results:**

Nineteen patients with peripheral precocious puberty were treated with acupuncture. After the 12-week acupuncture intervention, the serum LH level, breast Tanner stage, and TCM symptom scores decreased significantly compared with baseline (*P* < 0.05); the serum FSH and E2 level did not change significantly after the 12-week treatment (*P* > 0.05). After the 12-week follow-up, breast Tanner stage and TCM symptom scores decreased significantly compared with baseline (*P* < 0.05), and there was no statistical difference between serum sex hormone (LH, FSH, and E2) level and baseline level (*P* > 0.05). During the period of acupuncture treatment, no side effects or serious adverse events occurred.

**Conclusions:**

Acupuncture is effective in regulating the hormone level and controlling early development process. It may be a viable alternative to the treatment of peripheral precocious puberty in girls. However, further randomized controlled trials are needed.

## 1. Introduction

Childhood precocious puberty refers to the second sexual characteristics of girls before the age of 8 and boys before the age of 9 [1]. With the improvement in living standards, changes in dietary structure, and environmental exposure, the incidence of precocious puberty in children is also increasing year by year. Research studies show that the incidence of precocious puberty is higher in girls than in boys [2, 3]. Precocious puberty can lead to the emergence of secondary sexual characteristics in advance, which may lead to premature closure of epiphysis and short stature in adulthood, and also has a negative impact on children's psychological development. If the patients with rapid onset and progress are not intervened in time, peripheral precocious puberty may turn to central precocious puberty [4]. Therefore, a safe and effective intervention which can improve the symptoms of precocious puberty and slowing down the initiation time of development may be accepted by patients. After searching CNKI, VIP, WANFANG database, and PubMed, we found that there is no relevant clinical study on acupuncture treatment for peripheral precocious puberty. Therefore, this preliminary study summarizes the clinical data of outpatients with precocious puberty, explores the effect of acupuncture on reproductive hormone level and breast development of girls with precocious puberty, and provides clinical experience for intervention treatment of precocious puberty. This research shows that acupuncture for peripheral precocious puberty in girls may be a promising treatment.

## 2. Methods

### 2.1. Study Design

This was a prospective cohort study conducted in Acupuncture Department and Pediatrics Department of Growth and Development Clinic, Beijing Jishuitan Hospital. The project was approved by the Ethics Committee of Beijing Jishuitan Hospital in May, 2018 (NO. 201805-12). The duration of the study was 24 weeks. The acupuncture treatments were carried out by a senior acupuncturist with 10-year clinical experience. Participants were screened by pediatric experts with endocrinological background. Therapeutic outcome assessment and statistical analysis were conducted by research assistants and statisticians, who were both blinded to the treatment process. This study was based on the Helsinki Declaration.

Inclusion criteria are as follows: [5, 6]
  1. Secondary sexual characteristics appeared in advance (definition of age: breast development or pubic hair premature appearance before 8 years in girls)
  2. In gonadotropin-releasing hormone (GnRH) stimulation test, the peak value of LH was less than 3.3–5.0 IU/L, and the ratio of LH/FSH was less than 0.6
  3. Breast Tanner stage does not exceed stage III
  4. Gonadal ultrasound: ovarian volume was less than 1 ml, follicle diameter was less than 4 mm, and no ovarian cyst
  5. Bone age over actual age should not exceed one year
  6. Without medication or any other intervention treatment
Exclusion criteria are as follows: [7]
  1. Central precocious puberty
  2. Premature puberty caused by gonadal tumors, adrenal diseases, or other organic diseases
  3. Heterosexual precocious puberty in girls
  4. Chromosome abnormalities or hereditary diseases
  5. Received medication or any other intervention treatment

### 2.2. Acupuncture Therapy

Selection of acupuncture point and manual needle manipulation was based on TCM theory, clinical experience, and literature review analysis. Bilateral SP6 (Sanyinjiao) and ST29 (Guilai) were positioned according to the World Health Organization Standard Definitions [8]. SP6 is located in 3 *cun* above the medial malleolus and on the posterior border of the medial aspect of the tibia; ST29 is located in 4 *cun* below umbilicus and 2 *cun* lateral to the anterior midline of the abdomen. Stainless steel disposable sterile acupuncture needles (0.25 mm in diameter and 25 mm in length; Huacheng brand, Beijing Keyuanda Medical Appliances Co. Ltd) were used. After sterilization of the acupoints' skin, the needles were inserted vertically into the posterior edge of the tibia 3 *cun* above the medial malleolus (at SP6) with a depth of 15–20 mm and at bilateral SP6 and ST29, with a depth of about 15–20 mm. Needles were gently rotated until patients felt “de qi” sensation, which was a sore, numb, heavy, or swollen sensation. Then, the needles were retained for 20 minutes in the patients. A total of 24 sessions of acupuncture treatment were offered, twice a week for 3 months. Acupuncturist with 10-year clinical experience performed all the acupuncture procedures.

### 2.3. Outcome Measures

The primary outcome was the change in luteinizing hormone (LH), follicle-stimulating hormone (FSH) level, and breast Tanner stage from baseline to the end of treatment at week 12 and follow-up until week 24. The secondary outcomes included changes in estradiol (E2) level and traditional Chinese medicine (TCM) symptom scores (0 = none, 1 = mild, 2 = moderate, and 3 = severe) from baseline to week 12 (after treatment) and week 24 (follow-up). Girls with peripheral precocious puberty scored themselves according to their own subjective feelings. During the period of acupuncture treatment, all side effects and adverse events related to acupuncture were recorded, such as fainting, local infection, severe pain, hematoma, and broken needle [9].

### 2.4. Statistical Analysis

Statistical analysis was performed with the SPSS (version 16.0). Wilcoxon's signed rank test was used for quantitative data unfitting normal distribution and categorical data. Two-related sample tests were used for quantitative data fitting normal distribution to measure the difference between baseline and after treatment (week 12) and follow-up (week 24). Two-sided *P* < 0.05 was regarded as significant.

## 3. Results

### 3.1. Participants

**Figure 1:**
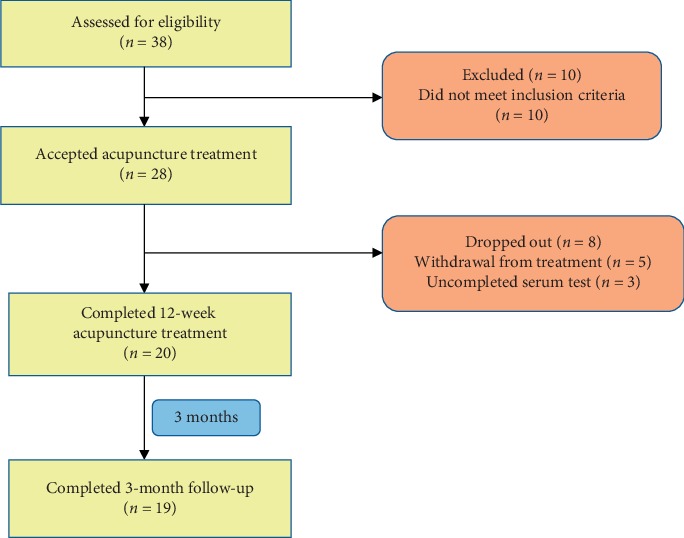
Flowchart of the study.

38 participants were recruited (Figure 1) from July 2017 to June 2018. Ten participants who did not meet inclusion criteria were excluded, five participants dropped out after accepting part sessions of acupuncture treatment, three participants did not complete serum test, the remaining 20 completed the acupuncture treatment, but 1 participant did not complete the follow-up. 19 participants completed the observation study after 12-week follow-up. The average age of these participants was 6.32 ± 1.42 years, with illness duration of 3.15 ± 3.31 months (Table 1). All the participants had breast development but without obvious areola pigmentation, one of them had premature pubarche together, and one had premature axillary hair together, and nineteen participants had never received acupuncture in the past.

### 3.2. Baseline Characteristics

Baseline characteristics of all the patients are provided in Table 1.

### 3.3. Serum Reproductive Hormone Level

After the 12-week treatment period, LH decreased from 0.24 ± 0.36 to 0.09 ± 0.07, the changes were significantly different compared with baseline (*P*=0.006), while FSH and E2 decreased from 3.19 ± 2.83 to 2.45 ± 0.71 and 54.81 ± 31.47 to 47.58 ± 19.69, respectively; the changes were not significantly different compared with baseline (*P*=0.546 and *P*=0.178). After the 12-week follow-up, LH, FSH, and E2 were 0.17 ± 0.18, 2.15 ± 0.63, and 64.3 ± 38.82, respectively, but there was no significant difference compared with baseline (*P*=0.26, *P*=0.059, and *P*=0.721, respectively) (Table 2).

### 3.4. Breast Tanner Stage

After the 12-week treatment and follow-up period, breast Tanner stage both improved compared with baseline (*P*=0.003 and *P*=0.008, respectively) (Table 3).

### 3.5. TCM Symptom Scores

After the 12-week treatment period, the TCM symptoms of breast tenderness, odor in mouth, palm hot flush, dry stool, and red tongue improved compared with baseline (*P*=0.001, *P*=0.008, *P*=0.001, *P*=0.001, and *P*=0.01, respectively), while hyperhidrosis did not improve significantly (*P*=0.102); after the 12-week follow-up, the TCM symptoms of breast tenderness, odor in mouth, palm hot flush, and red tongue improved compared with baseline (*P*=0.001, *P*=0.003, *P*=0.001, and *P*=0.013, respectively), but hyperhidrosis and dry stool did not improve significantly (*P*=0.059 and *P*=0.132). The total score of TCM symptoms both improved after 12-week treatment (*P*=0.01) and 12-week follow-up (*P*=0.01), compared with baseline (Table 4).

### 3.6. Safety

In this study, there were no serious adverse events. Only 2 cases of abdominal mild subcutaneous hematomas occurred after treatment, but disappeared within one week. Needling pain occurred after treatment in 2 cases and disappeared within 12 hours. No other discomfort and side effects were found. Acupuncture was safe in the treatment of precocious puberty.

## 4. Discussion

The initiation of puberty in children depends on genetic and neuroendocrine factors and is affected by nutritional, exercise, and exposure to environmental chemicals [10–12]. Precocious puberty is a disease associated with puberty development, which affects the physical and mental health of children [13], causes the incidence rate of obesity to increase, and increases the risk of cardiovascular disease and metabolic diseases in children after adulthood [14]. The etiology of some children with precocious puberty is not clear, and about 15% of children with precocious puberty can develop into central precocious puberty by inducing the rapid maturation of hypothalamus pituitary gonad axis [15, 16]. Therefore, this disease needs to be highly concerned by parents and doctors. At present, in addition to lifestyle intervention and regular clinical observation, there is no unified and effective treatment method to control the puberty development process of children with nonorganic peripheral precocious puberty.

The aim of this study was to explore whether acupuncture could control the early development of breasts and regulate the level of sexual hormones in girls with precocious puberty and improve the related symptoms, so as to slow down the sexual development process of girls with precocious puberty. The results showed that, after 12 weeks of treatment, the breast Tanner stage and LH level decreased significantly and the related TCM symptoms also improved significantly. After 12 weeks of follow-up, there were also differences in breast Tanner stage and TCM symptoms compared with baseline.

The first manifestation of puberty development in girls is breast development. Breast Tanner stage is a direct indicator of the development of girls' secondary sexual characteristics [17]. Generally, there are no other signs of puberty development in early breast development, such as linear growth acceleration and bone age advance, but some children will still develop into progressive precocious puberty. The results of this study showed that acupuncture could significantly improve breast Tanner stage in girls with precocious puberty, indicating that acupuncture was effective in controlling the early development of girls' breast, which reduced the risk of progressive precocious puberty.

The initiation of female puberty is first manifested by the increase in FSH level. FSH is related to the development and maturation of female follicles, which can reflect the initiation of gonadal axis. In this study, the mean value of FSH in children decreased after 12-week treatment and follow-up, which indicated that acupuncture played an inhibitory role in the regulation of follicular development and maturation, but there was no significant difference compared with baseline; whether this was related to acupuncture frequency and course of treatment remains to be further studied.

LH is the gonadotropin secreted by the pituitary gland, which is related to the initiation of gonadal axis and the development of secondary sexual signs. The increase in LH level is considered as a powerful marker of gonadal axis activation. The basic LH level is the best screening method for the diagnosis of central precocious puberty, which is lower than 0.1 IU/L, indicating that it is in the prepubertal stage [18]. The results of this study showed that, after 12 weeks of acupuncture treatment, the LH level of children decreased and the mean value was lower than 0.1 IU/L; the difference was statistically significant, suggesting that acupuncture treatment could reduce the LH level of girls with precocious puberty. The decrease in LH was helpful to improve the breast Tanner stage, and the results of the two indexes in this study were consistent. After 12-week follow-up, the mean value of LH was still lower than the baseline level, suggesting that there was no significant progress in the sexual development of children, but there was no significant difference compared with the baseline, suggesting that acupuncture could control the sexual development process, but the long-term effect might not be obvious.

Although there are few studies on the effects of acupuncture on children's sexual development and secondary sexual signs, there are relevant clinical and basic researches on the effects of acupuncture on adult women's reproductive endocrine function [19, 20]; literature studies have shown that acupuncture treatment can reduce the serum FSH and LH levels of adult women, improve the E2 level of women, and improve menstrual cycle and ovarian function [21, 22]; acupuncture has been applied to primary ovarian dysfunction [23], polycystic ovarian syndrome [24], menopausal syndrome [25], dysmenorrhea [26], and other female reproductive endocrine-related diseases. Although the mechanism of acupuncture on female reproductive endocrine system is not very clear at present, it has been found that acupuncture can regulate some neurotransmitters and neuropeptides, such as *β*-endorphin, dopamine, and prostaglandin [27–29] and then affect the hypothalamic pituitary gonadal axis, thus regulating the level of serum sex hormones [30]. The results of this study suggested that acupuncture had a positive effect on the sexual hormone level of precocious puberty children, which could reduce the level of LH and FSH, indicating that acupuncture could control the development process of precocious puberty to some extent. Acupoint has the characteristics of bidirectional benign regulation, which can produce benign regulation on different functional states of the body. The acupuncture's regulation mechanism on children may be similar to that of adult women, by regulating related neurotransmitters of neuro-endocrine-immune network; it can affect children's gonadal axis, regulate their growth and development process, and improve children's secondary sexual signs. The effect and mechanism of acupuncture on children's sexual development may involve multilevel, multitarget, and multichannel, which need further observation and research.

Estradiol (E2) is the main component of estrogen, which is mainly secreted by ovarian granulosa cells and participates in the development of female reproductive organs and secondary sexual signs. The estrogen level of girls is easily affected by exogenous intake or environmental chemical exposure [31]; the precocious puberty caused by exogenous estrogen intake is mainly manifested as breast enlargement, obvious pigmentation of areola and vulva, and significant increase in blood estradiol. In this study, all the children only had breast enlargement and no areola and vulvar pigmentation, so the effect of exogenous hormones could be excluded. Moreover, the level of E2 is mainly affected by the growth of follicles and also easily affected by FSH and inhibin B. The variation in estrogen level is large. Therefore, E2 level is not the main observation index of acupuncture effect in this study. The results of this study suggested that there was no significant difference in E2 level after 12-week treatment and follow-up compared with the baseline level, and the average estradiol level of girls was within the normal range, indicating that the early breast development of girls was not parallel to the increase in estradiol level [32].

In this study, the girls with precocious puberty did not have a clear history of exposure to exogenous hormones and had no organic diseases related to precocious puberty [33, 34]. The common clinical TCM symptoms are breast pain or tenderness, bad smell in the mouth, hot palm, sweating, dry stool, and red tongue. According to the theory of TCM, the main pathogenesis of precocious puberty is deficiency of kidney yin and liver depression into fire [35]. The therapeutic principles of TCM are nourishing kidney yin, relieving liver depression, and reducing fire [36]. Some studies show that Chinese herbal medicine has certain effect on central precocious puberty [37], but medicine has the risk of liver and kidney function damage and other side effects. Therefore, this study attempts to evaluate the efficacy and safety of acupuncture intervention. The results showed that, after 12-week acupuncture treatment and follow-up, the TCM symptom scores of the children were significantly improved; the breast tenderness was significantly alleviated, and odor in mouth, hot palm, dry stool, and red tongue were also improved to varying degrees; there were no serious adverse events and side effects during acupuncture treatment, which indicated that acupuncture was effective and safe in treating precocious puberty. The improvement in acupuncture on TCM symptoms might be related to the regulation of gonadal axis or sex hormone level. Bilateral SP6 (Sanyinjiao) and ST29 (Guilai) were selected to treat children's precocious puberty in the study, which were in line with the characteristics of acupoints and the TCM pathogenesis of precocious puberty, and followed the principle of syndrome differentiation and treatment in traditional Chinese medicine. SP6 is the intersection point of three meridians of liver, spleen, and kidney; ST29 belongs to the spleen meridian. According to literature research and clinical experience, the two acupoints are commonly used to treat reproductive endocrine diseases and regulate the level of sexual hormones.

In a word, after acupuncture treatment, breast Tanner stage of precocious girls was improved, serum FSH and LH levels were decreased, and TCM symptoms were improved in varying degrees. Therefore, acupuncture intervention can effectively control the sexual development process of girls with peripheral precocious puberty, slow down breast development, reduce the level of sex hormone, and improve TCM symptoms, and it is safe. However, as a prospective preliminary study, the sample size of this study is relatively small, the follow-up time is short, and the lack of control group may lead to biased results, so it is necessary to increase the sample size; randomized controlled studies are needed to further confirm the therapeutic effect of acupuncture on girls' peripheral precocious puberty.

## 5. Conclusions

This preliminary study suggests that acupuncture can slow down the sexual development process of girls with peripheral precocious puberty, and it is safe. Its efficacy and safety still need to be further confirmed by randomized controlled trials.

## Figures and Tables

**Table 1:** Baseline characteristics of the 19 patients.

Characteristics	Mean ± SD or *N*
Ages (years)	6.32 ± 1.42
Minimum age (years)	4.25
Maximum age (years)	8.33
Duration of PPP (months)	3.15 ± 3.31
Maximum duration (months)	12
Minimum duration (weeks)	1

**Table 2:** The levels of LH, FSH, and E2 (*x* ± *s*).

Outcomes	*N*	Baseline	12-week	After follow-up	*P*_1_ value	*P*_2_ value
LH (IU/L)	19	0.24 ± 0.36	0.09 ± 0.07	0.17 ± 0.18	0.006^*∗*^	0.26
FSH (IU/L)	19	3.19 ± 2.83	2.45 ± 0.71	2.15 ± 0.63	0.546	0.059
E2 (pmol/L)	19	54.81 ± 31.47	47.58 ± 19.69	64.3 ± 38.82	0.178	0.721

Wilcoxon's signed-rank test; ^*∗*^*P* < 0.05. *P*_1_ value indicating 12-week treatment compared with baseline; *P*_2_ value indicating follow-up compared with baseline.

**Table 3:** Breast Tanner stage.

Breast Tanner	*n*	Baseline			12-week			After follow-up			*P*_1_ value	*P*_2_ value
		I	II	III	I	II	III	I	II	III		
Left	19	2	12	5	10	7	2	10	7	2	0.003^*∗*^	0.008^*∗*^
Right	19	3	10	6	10	8	1	10	7	2		

Chi-square test; ^*∗*^*P*_1_ < 0.05 indicating 12-week treatment compared with baseline; ^*∗*^*P*_2_ < 0.05 indicating follow-up compared with baseline.

**Table 4:** TCM symptom scores (*x* ± *s*).

Item assessed	*N*	Baseline	12-week	After follow-up	*P*_1_ value	*P*_2_ value
Breast tenderness	19	2.16 ± 0.69	0.47 ± 0.61	1.32 ± 0.58	0.001^*∗*^	0.001^*∗*^
Odor in mouth	19	1.16 ± 0.83	0.63 ± 0.60	0.68 ± 0.82	0.008^*∗*^	0.003^*∗*^
Palm hot flush	19	2.16 ± 0.83	1.58 ± 0.61	1.47 ± 0.70	0.001^*∗*^	0.001^*∗*^
Hyperhidrosis	19	1.74 ± 0.87	1.53 ± 0.90	1.47 ± 0.77	0.102	0.059
Dry stool	19	1.47 ± 0.84	0.84 ± 0.60	1.21 ± 0.71	0.001^*∗*^	0.132
Red tongue	19	1.68 ± 0.67	0.95 ± 0.52	1.21 ± 0.63	0.01^*∗*^	0.013^*∗*^
Total score	19	10.37 ± 2.01	7.0 ± 1.56	7.37 ± 1.95	0.01^*∗*^	0.01^*∗*^

Wilcoxon's rank sum test; ^*∗*^*P*_1_ < 0.05 indicating 12-week treatment compared with baseline values; ^*∗*^*P*_2_ < 0.05 indicating follow-up compared with baseline values.

## Data Availability

The measurement and classification data used to support the findings of this study are included within the supplementary information file.

## Abbreviations

LH: Luteinizing hormone
FSH: Follicle-stimulating hormone
E2: Estradiol
PPP: Peripheral precocious puberty
CPP: Central precocious puberty
TCM: Traditional Chinese medicine
GnRH: Gonadotropin-releasing hormone.

## Supplementary Materials

Supplementary Materials The supplementary materials submitted along with the manuscript mainly involved the observation indexes, including serum reproductive hormone (LH, FSH, and E2), breast Tanner stage, and TCM symptoms (baseline, 12-week treatment, and 12-week follow-up).
